# Infection of a Total Femur Megaprosthesis: A Retrospective Study of Clinical and Functional Outcomes

**DOI:** 10.7759/cureus.106870

**Published:** 2026-04-12

**Authors:** David Mayorga-Naranjo, Amparo Ortega-Yago, Daniel Bonete-Lluch, Ignacio Baixauli-García, Francisco Argüelles-Linares, José Baeza-Oliete

**Affiliations:** 1 Department of Orthopaedic Surgery and Traumatology, Hospital Universitari i Politècnic La Fe, Valencia, ESP; 2 Department of Orthopaedics and Traumatology, Hospital Universitari i Politècnic La Fe, Valencia, ESP; 3 Department of Orthopedics and Traumatology, Hospital Universitari i Politècnic La Fe, Valencia, ESP

**Keywords:** complications, megaprosthesis, prosthetic joint infection, reconstruction, total femur replacement

## Abstract

Introduction: Infection associated with total femur megaprostheses represents an uncommon and highly complex clinical scenario, typically occurring in patients with multiple previous surgeries and massive bone loss. Evidence regarding outcomes in non-oncologic indications remains limited.

Methods: A retrospective study was conducted, including patients treated with total femur megaprosthesis for prosthetic joint infection at Hospital Universitari i Politècnic La Fe, a tertiary referral center in Valencia, Spain, between 2016 and 2023. Clinical, microbiological, and outcome data were collected from medical records. An exploratory analysis was performed, comparing patients with favorable and unfavorable outcomes using non-parametric tests.

Results: Ten patients were included, with a mean age of 69 years and a median of six previous surgical procedures. Comorbidities included diabetes mellitus (30%), immunosuppression (40%), and rheumatic disease (20%). The microbiological spectrum was heterogeneous, with *Staphylococcus epidermidis *identified in 50% of cases and polymicrobial infections in 40%. *Candida* species and *Pseudomonas aeruginosa* were each identified in 20% of patients. Complications occurred in seven patients (70%), most commonly reinfection and instability. At the final follow-up, eight patients (80%) required walking aids. Two patients (20%) died from causes not directly related to infection.

Conclusions: Total femur megaprosthesis in septic settings is a salvage procedure that may allow limb preservation in highly complex patients, although it is associated with a high complication rate and limited functional outcomes. Its use should be restricted to selected cases and specialized centers. Further multicenter studies are needed to optimize patient selection and management strategies.

## Introduction

Total femur megaprosthesis is a salvage reconstructive option used in patients with massive bone stock loss and failure of multiple previous reconstructions, both in oncologic and, increasingly, non-oncologic indications. In recent years, its use has expanded in non-tumoral scenarios such as complex periprosthetic fractures, nonunion, severe mechanical failure, and prosthetic joint infection, although it remains a technique generally reserved for highly specialized centers [1-4].

While the incidence of infection following primary hip or knee arthroplasty has been reported to be approximately 1-2% in large international series, infection rates in megaprostheses are considerably higher, ranging from 15% to 40% depending on anatomical location, implant length, and host characteristics [2-4]. Reconstructions involving long-segment replacements, such as total femur replacement, appear to be associated with particularly high risks of infection, mechanical failure, and reoperation [2,5,6].

In septic scenarios, a total femur megaprosthesis is considered a salvage procedure after multiple surgical interventions for hip or knee prosthetic joint infection, infected periprosthetic fractures, or nonunion. These cases often coexist with massive bone defects, compromised soft tissues, high bacterial burden, and significant comorbidity. Available series report high complication, reinfection, and reoperation rates, with a non-negligible risk of amputation. However, infection control and implant survival may be acceptable in selected cases, particularly when multi-stage exchange strategies are employed [6-9].

Evidence specifically addressing infection in total femur replacement is scarce, and published series frequently group different femoral segment replacements or mix oncologic and non-oncologic indications. At the national level, studies on total femur prostheses in non-oncologic patients are limited and include few cases in which infection is the primary indication [1,5,10]. Therefore, the aim of this study was to evaluate clinical outcomes following total femur megaprosthesis implantation for prosthetic joint infection in a non-oncologic setting. Secondary objectives were to characterize the microbiological profile, assess complication rates, and describe functional outcomes and survival.

## Materials and methods

Study design

A retrospective descriptive study was performed, including patients treated at Hospital Universitari i Politècnic La Fe, a national referral center for osteoarticular infections in Valencia, Spain, who underwent total femur megaprosthesis implantation due to infectious causes. All procedures were performed between 2016 and 2023, comprising a seven-year study period.

In accordance with institutional policy, formal Ethics Committee approval was waived due to the retrospective design and anonymized nature of the data.

Inclusion criteria were patients older than 18 years, treated with total femur megaprosthesis for infectious indications, with a minimum follow-up of two years or until death. Exclusion criteria included patients under 18 years, those treated with other types of prostheses, cases related to oncologic disease management, or follow-up shorter than two years in living patients.

The following variables were collected: age at diagnosis, sex, body mass index (BMI), associated comorbidities (rheumatic disease, immunosuppressive therapy/immunosuppression, and diabetes mellitus), presence of a previous total hip arthroplasty, number of previous surgical procedures, causative microorganism, type of total femur megaprosthesis used, complications, need for walking aids, and mortality. Data were retrospectively obtained from electronic medical records and microbiology databases and subsequently anonymized prior to analysis. Data collection was performed by the study investigators and cross-checked to ensure accuracy and completeness.

Diagnostic definition

Prosthetic joint infection was diagnosed according to the European Bone & Joint Infection Society criteria, considering infection confirmed in the presence of one or more major criteria (sinus tract, two positive cultures with the same microorganism) or at least three minor criteria (elevated C-reactive protein, local signs, compatible histological findings, or a single positive culture) [11].

Outcome definition

For the exploratory analysis, outcomes were defined a priori. A favorable outcome was defined as infection eradication with implant retention at last follow-up, without the need for further surgery due to infection. An unfavorable outcome included reinfection, need for additional surgery due to infection, implant removal, disarticulation, or infection-related death. Isolated mechanical complications, such as hip dislocation successfully managed without evidence of recurrent infection, were not considered unfavorable outcomes, as the primary objective of the study was to evaluate infection control.

Follow-up

Postoperative follow-up was performed at two weeks, one month, three months, six months, one year, and annually thereafter, with serial radiographs. Complications during follow-up were recorded, including reinfection, and monitored through laboratory tests in collaboration with Infectious Diseases. Functional data were also collected.

Statistical analysis

Statistical analysis was performed using R version 4.4.0 (R Foundation for Statistical Computing, Vienna, Austria). Continuous variables were reported as mean ± standard deviation (SD) or median (interquartile range, IQR). Due to the small sample size, non-parametric tests were used.

Continuous variables were compared using the Mann-Whitney U test and categorical variables using Fisher’s exact test. Statistical significance was set at p < 0.05. Microbiological distribution was represented using a pie chart.

For statistical purposes, patients were classified as having a favorable outcome if no complications occurred during follow-up, and an unfavorable outcome if any complication was recorded.

Kaplan-Meier curves were generated for overall survival using follow-up time as the time variable and death from any cause as the event; curve comparison was performed using the log-rank test. Due to the low number of events, no robust multivariate models were conducted.

## Results

Ten patients undergoing total femur megaprosthesis implantation for prosthetic joint infection were included. The mean age was 69.0 ± 9.4 years (median 67.0 (66.0-76.0)). Five patients were male (50%), and five were female (50%). The mean BMI was 26.2 ± 5.1 kg/m² (median 26.6 (23.1-30.1)). The mean number of previous surgical procedures prior to implantation was 6.6 ± 3.0 (median 6.0 (5.0-6.8)). Follow-up was available in nine patients and averaged 5.7 ± 2.2 years (median 6.0 (4.0-6.0)). Regarding comorbidities, diabetes mellitus was present in three patients (30%), immunosuppressive treatment/immunosuppression in four (40%), and rheumatic disease in two (20%).

For the exploratory analysis, outcomes were defined as follows: a favorable outcome was considered infection eradication with implant retention at the last follow-up, without the need for further surgery for infection. Unfavorable outcomes included reinfection, need for further surgery due to infection, implant removal, disarticulation, or infection-related death. Isolated mechanical complications, such as pure hip dislocation successfully managed without evidence of recurrent infection, were not classified as unfavorable outcomes, as the primary objective of the study was to evaluate infection control rather than mechanical failure.

In the exploratory comparison between favorable (n = 5) and unfavorable (n = 5) outcome groups, no statistically significant differences were observed in age, sex, BMI, number of previous interventions, follow-up duration, diabetes mellitus, immunosuppression, or rheumatic disease (Table 1).

**Table 1:** Exploratory comparison between favorable and unfavorable outcome groups.

Variable	Favorable outcome (n = 5)	Unfavorable outcome (n = 5)	p value
Age (years)	76.0 (71.0–79.0)	66.5 (61.8–67.0)	0.24
Sex (male), n (%)	2 (40%)	3 (60%)	0.86
BMI (kg/m²)	25.0 (22.5–26.0)	30.4 (27.2–31.7)	0.18
Previous surgical procedures (number)	5.0 (4.0–9.0)	6.0 (6.0–6.0)	0.91
Follow-up duration (years)	6.0 (5.0–9.0)	5.0 (3.8–6.0)	0.79
Diabetes mellitus, n (%)	0 (0%)	3 (60%)	0.47
Immunosuppression, n (%)	0 (0%)	4 (80%)	0.50
Rheumatic disease, n (%)	0 (0%)	2 (40%)	0.80

Among the nine patients with available data, five (55.6%) had a previous total hip arthroplasty, isolated or associated with other fixation systems. Indications for megaprosthesis were predominantly hip or knee prosthetic joint infection, sometimes associated with periprosthetic fracture or nonunion, in a highly complex setting (median of six previous procedures).

Multidisciplinary management and therapeutic strategy

All patients were managed within a multidisciplinary osteoarticular infection board involving Orthopaedic Surgery, Microbiology, Plastic Surgery, Infectious Diseases, and Radiology. Treatment strategies were individualized based on patient characteristics, comorbidities, and infection features.

Surgical management

Surgical treatment included one-stage or two-stage exchange procedures, as well as DAIR in selected cases. In two-stage procedures, the first stage involved prosthesis removal, extensive debridement, microbiological sampling, and placement of an antibiotic-loaded spacer. The second stage consisted of spacer removal, repeat debridement, and implantation of the definitive total femur megaprosthesis (OSS Total Femur System, Zimmer Biomet, Warsaw, USA). The specific surgical approach and reconstruction strategy were adapted to each patient according to local and systemic conditions.

Antibiotic treatment

Antibiotic therapy was managed by the Infectious Diseases Department. Empirical treatment was initiated in cases without prior microbiological identification and subsequently adjusted according to antimicrobial susceptibility testing. All patients received an initial intravenous regimen followed by oral therapy, with a minimum total duration of six weeks. Treatment duration and regimen varied depending on the causative microorganism and surgical strategy.

Microbiology

The microbiological spectrum was heterogeneous, including Gram-positive cocci, Gram-negative bacilli, and fungi. *Staphylococcus epidermidis* was involved in five of 10 cases (50%), either as a monomicrobial infection or as part of polymicrobial infections. *Candida *species were isolated in two patients (20%), and *Pseudomonas aeruginosa* in two cases (20%); remaining isolates included *Enterobacteriaceae* and *Enterococcus faecalis*. Polymicrobial infections were identified in 40%, with combinations of coagulase-negative staphylococci and Gram-negative bacilli.

Microbiological samples were processed according to standard laboratory protocols. Identification of microorganisms and antimicrobial susceptibility testing were performed in the Microbiology Department following established clinical guidelines (CLSI or equivalent standards). Targeted antibiotic therapy was guided in all cases by susceptibility testing results.

Implant type

In six patients (60%), a Zimmer total femur megaprosthesis was used, and in two (20%), a Link megaprosthesis. In two cases (20%), the specific system could not be determined from medical records.

Complications

Seven patients (70%) experienced at least one complication related to the megaprosthesis or infection. Reported complications included reinfection, recurrent hip dislocation, periprosthetic fracture, and adjacent soft tissue/bone osteomyelitis, with coexistence of multiple complications in some cases. Management strategies included DAIR, one- or two-stage revision, hip disarticulation, and soft tissue coverage using musculocutaneous flaps depending on severity and patient condition (Table 2).

**Table 2:** Complications and treatment performed.

Patient	Age	Sex	Complication	Treatment performed
1	67	Female	Reinfection associated with recurrent dislocations	Hip disarticulation
2	79	Male	Dislocation	Reduction
3	66	Female	Dislocation	Reduction
4	49	Male	Reinfection	DAIR
5	67	Male	Reinfection	Two-stage revisión
6	76	Female	Periprosthetic fracture	Immobilization with splint
7	80	Female	Osteomyelitis	Debridement and flap coverage

Functional outcome

At final follow-up, eight of ten patients (80%) required walking aids. Among cases with specified assistance, wheelchair use predominated (three cases), followed by crutches (three cases) and a walker (two cases). In two medical records, the type of aid was not specified, although an overall significant functional limitation was documented.

Survival (exploratory analysis)

**Figure 1:**
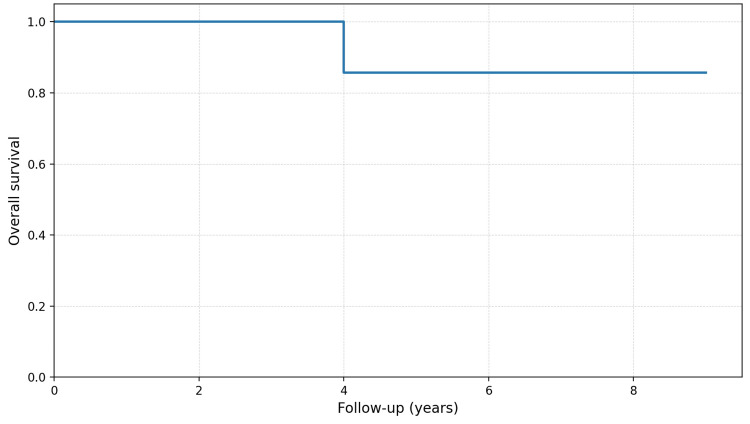
Kaplan-Meier survival curve of the cohort.

During follow-up, two patients (20%) died from causes not directly attributable to prosthetic joint infection (cardiac complications in one case and respiratory complications in the other). Kaplan-Meier analysis estimated a cumulative survival of 85.7% at the end of available follow-up (Figure 1).

Given the limited sample size and number of events, survival analysis should be considered exploratory. No statistically significant differences were observed according to diabetes mellitus (log-rank p = 0.53). For immunosuppression, a non-significant trend toward worse survival was observed (log-rank p = 0.11).

## Discussion

Total femur megaprosthesis in a septic setting represents an extreme reconstructive strategy reserved for salvage situations in patients with severe local and systemic complexity. Its main goal is to avoid ablative procedures and preserve a functional limb, albeit at the cost of a high risk of complications. Therefore, outcomes should be interpreted as those of a salvage procedure rather than a conventional definitive reconstruction [1,2].

In our series of 10 patients treated with total femur megaprosthesis in a septic setting, patients were elderly (mean age 68 years), with significant comorbidity (20% rheumatic disease, 30% diabetes, and 40% immunosuppression or immunocompromised) and a very high number of previous surgeries (median of six procedures). This profile is consistent with other non-oncologic megaprosthesis series, where most procedures are performed in older, frail patients with multiple revisions [1,2,10].

The complication rate in our cohort (70%) was high but consistent with published literature on megaprostheses in prosthetic joint infection, where global failure rates (including reinfection, dislocation, mechanical loosening, fracture, and soft tissue failure) range from 20% to 40%. Reinfection and dislocation, often combined, are in line with other reports identifying instability and recurrent infection as the most frequent failure modes after megaprosthesis implantation in previously infected patients [5,8-10,12].

The highly heterogeneous microbiological spectrum, with a significant proportion of *Staphylococcus epidermidis*, Gram-negative bacilli, and fungi (*Candida albicans* and *C. glabrata*), is also consistent with recent series on megaprosthesis infections and oncologic patients. Coagulase-negative staphylococci, polymicrobial infections, and a relevant proportion of fungal infections have been increasingly reported. Although *Candida* prosthetic joint infections are uncommon, they are associated with higher treatment failure rates, more revision surgeries, and frequently more aggressive strategies such as resection arthroplasty or disarticulation. The presence of two fungal infections in such a small cohort suggests that patients requiring total femur replacement represent a subgroup with a particularly high risk of complex infections [5,6,13].

From a functional perspective, most patients required walking aids at the final follow-up (80%), predominantly wheelchairs, crutches, or walkers. Although this may appear unfavorable, it must be interpreted within the context of limb salvage after multiple surgeries, extensive bone and soft tissue destruction, and, in many cases, where the realistic alternative would have been amputation. Several studies have shown that despite functional limitations and high complication rates, femoral megaprostheses allow limb preservation and acceptable function in a substantial proportion of patients compared to disarticulation or amputation [7,9,10,12,14-17].

In our series, the 20% mortality rate during follow-up, apparently due to causes not directly attributable to prosthetic joint infection, is consistent with the high comorbidity burden of these patients and with other reports describing significant mortality and an amputation risk of up to 30-35% in megaprosthesis infection settings [18]. Although absolute numbers are small due to sample size, these findings highlight the severity of these conditions and the importance of appropriate patient selection and realistic preoperative counseling.

Our results support the concept that total femur megaprosthesis in septic settings should be considered a salvage procedure reserved for selected cases and requiring multidisciplinary coordination among orthopaedic surgery, infectious diseases, microbiology, and plastic surgery. Multi-stage exchange strategies appear to offer better infection control rates compared to DAIR, although at the cost of additional surgeries and increased patient burden. In our cohort, multiple strategies were employed (DAIR, staged revision, disarticulation) depending on local and systemic status, reflecting the absence of a universal algorithm and the need for individualized management.

This study has several limitations. First, the sample size is very small (n = 10), which limits generalizability and prevents robust statistical analysis or identification of prognostic factors. However, this reflects the rarity and complexity of total femur megaprosthesis in septic settings. Second, the retrospective single-center design introduces potential selection and information bias, as data were obtained from medical records, and some variables may be incomplete or inconsistently reported.

The cohort was heterogeneous, including different underlying conditions (hip or knee prosthetic joint infection, periprosthetic fracture, nonunion), a wide range of previous surgical procedures, and diverse microbiological profiles, including fungal infections. This heterogeneity limits comparability between cases and with other published series. In addition, functional outcomes were assessed descriptively without the use of standardized scoring systems, which limits objective comparison with other studies.

Despite these limitations, this study has several strengths. It focuses on a rare and highly complex clinical scenario, with all cases performed for non-oncologic septic indications, which are underrepresented in the literature. The relatively long follow-up provides meaningful insight into mid-term outcomes. Furthermore, the study offers a detailed clinical and microbiological characterization of this challenging patient population, reflecting real-world practice in a specialized referral center. These findings contribute to the current understanding of total femur megaprosthesis as a salvage procedure and may help guide clinical decision-making and future research.

## Conclusions

Total femur megaprosthesis in septic settings is a salvage procedure performed in highly complex patients with multiple previous surgeries. The microbiological spectrum was heterogeneous, including coagulase-negative staphylococci, Gram-negative bacilli, and fungal infections. Although it enables limb preservation, it is associated with a high complication rate and limited functional outcomes. Its indication should be restricted to selected patients and centers with multidisciplinary expertise. Larger multicenter studies are required to optimize indications, surgical strategies, and outcomes.
